# Emotional Intelligence Components as Predictors of Engagement in Nursing Professionals by Sex

**DOI:** 10.3390/healthcare8010042

**Published:** 2020-02-22

**Authors:** María del Mar Molero Jurado, María del Carmen Pérez-Fuentes, Ana Belén Barragán Martín, José Jesús Gázquez Linares, Nieves Fátima Oropesa Ruiz, María del Mar Simón Márquez

**Affiliations:** 1 Department of Psychology, University of Almería, 04120 Almería, Spain; mmj130@ual.es (M.d.M.M.J.); abm410@ual.es (A.B.B.M.); foropesa@ual.es (N.F.O.R.); msm112@ual.es (M.d.M.S.M.); 2 Department of Psychology, Universidad Autónoma de Chile, Santiago 4780000, Chile

**Keywords:** engagement, emotional intelligence, nursing, sex

## Abstract

Engagement of nursing professionals is related to their psychological wellbeing, and therefore, emotional intelligence acts as a predictor. The purpose of this study was to analyze the explanatory value of the dimensions of emotional intelligence in engagement in both sexes, as well as the conditional effect of interaction of sex as a moderating variable. The sample was comprised of 2126 nurses with a mean age of 31.66 years. The Utrecht Work Engagement Scale and the Brief Inventory of Emotional Intelligence for Senior Citizens (EQ-i-20M) were used for their evaluation. The results revealed the existence of significant differences in engagement depending on the sex of the nursing professionals. Furthermore, this study showed that the interpersonal component of emotional intelligence is the predictor of engagement of female professionals, while mood and the interpersonal dimensions have a higher predictive value of engagement in males. Finally, this study was able to emphasize the sex differences found along with the importance of the role emotional intelligence dimensions have in engagement levels, which must be taken into account when designing intervention programs to improve engagement and promote participation of nursing professionals in their workplace.

## 1. Introduction

Wellbeing is related to medical attention and job productivity [1,2], so it has important implications for the individual and the community. Nursing professionals are the largest group in the field of healthcare, since they are much in demand with the increasing incidence of diseases requiring their work [3]. Coping with these situations requires professionals committed to their job and daily care of patients, and this is called engagement. Engagement is described as a satisfactory positive mental state which is linked to work and is characterized by vigor, dedication, and absorption [4,5]. Vigor refers to time and effort dedicated to doing the job, dedication refers to the extent of one’s involvement in work on the job, and absorption is becoming completely immersed in one’s work, so that time seems to go faster [6].

Engagement is a positive influence on the work of healthcare personnel, and increases their efficacy [4] and performance, and has a favorable effect on attention [7]. On the contrary, a low level of engagement with one’s work can cause unfavorable situations and the presence of symptoms, such as burnout [8,9], as the emotional demand of the patients, work shifts, and organizational and personal conflicts make nursing one of the most stressful professions [10]. Strongly engaged nurses show dedication to their work, which does not mean they do not consider the problems that make it hard to remain committed [11]. Moreover, as their job satisfaction increases, so does their self-efficacy and engagement [12]. Studies have found that engagement has a mediating role in relationships with other variables, such as personal and work resources, in the intention of quitting [13], and job security, as one of the factors determining stronger engagement in one’s work [14]. Engagement and self-efficacy also positively predict job performance, and therefore more personal initiative [15]. However, the relationship between self-efficacy and engagement is not only found in the work setting. A relationship between perception of self-efficacy and engagement has also been found in academic settings in health sciences students [16]. Similarly, motivation exerts a mediating role in engagement, as confirmed in a sample of athletes, where high levels of self-determined motivation increased engagement [17].

Engagement of nursing professionals is further linked to their psychological wellbeing, as shown in several studies on the role of emotions with regard to the professional variables of healthcare workers [18]. For example, emotional resources generate positive attitudes and skills for developing engagement, motivating workers to make their best effort and put the most energy into the job [19], thus preceding wellbeing and good job satisfaction [20,21]. In addition, in the field of emotions, emotional intelligence acts as a predictor of wellbeing, and therefore, of engagement [22]. This study therefore intended to find out the role of the emotional intelligence dimensions in engagement in a sample of nurses.

### Relationship between Emotional Intelligence and Engagement

The level of emotional intelligence influences personal achievement of healthcare professionals [23] and the general population [24], as strong emotional work has a direct effect on increasing engagement [25]. It has been found that in nursing personnel, there are significant differences between the sexes in all the dimensions of engagement, where women had higher scores than men [26,27]. These authors have emphasized the high levels of emotional intelligence of women in interpersonal and intrapersonal components. Nurses who have high levels of emotional and behavioral self-regulation can believe in their ability to achieve job goals and feel more involved, so it is likely that the ability to regulate emotions on the job improves engagement [28]. Azimi, AsgharNejad, Kharazi, and Khoei [29] found higher scores in the stress management, mood, and adaptability dimensions in their group of male nurses.

Akhtar, Boustani, Tsivrikos, and Chamorro-Premuzic [30] argued that there is a correlation between mood regulation and the components of engagement in the healthcare context, and that therefore, the dimensions of emotional intelligence act as predictors of engagement. A study by Zhu, Liu, Guo, Zhao, and Lou [31] found that emotional intelligence is a significant predictor of work engagement in a group of nurses, with an indirect effect on engagement through organizational justice. According to García-Sierra, Fernández-Castro, and Martínez-Zaragoza [11], dedication is associated with the individual’s mood, and in women nurses, work becomes a source of personal growth for them. These authors emphasized the absorption component as unrevealing for nursing, given the sample characteristics. Pérez-Fuentes, Molero, Gázquez, and Oropesa [27] showed that the interpersonal factor of emotional intelligence was one of the most predictive variables and that mood was a mediating variable in the three components of engagement.

In spite of the importance of emotional intelligence as a predictor variable in the development of engagement in professionals, there is a limitation in the literature concerning differences in sex with respect to the role of each of its dimensions.

Therefore, the objective posed in this study was to analyze the explanatory value of the dimensions of emotional intelligence in engagement, as well as the conditional effect of interaction of sex as a moderating variable.

Based on empirical evidence, the following hypotheses are posed: (1) There are differences in engagement by sex; (2) mood and interpersonal dimensions will be the best predictors of engagement of male professionals; (3) the interpersonal component of emotional intelligence will be the strongest predictor of engagement in female professionals; (4) the interpersonal component of emotional intelligence will show the most explanatory value for the vigor dimension of engagement in female professionals.

## 2. Materials and Methods 

### 2.1. Participants

The original sample was made up of 2218 active nurses in Andalusia (Spain) selected at random from several centers. Ninety-two subjects were removed from the sample because they did not complete the questionnaire (32 subjects) or because of random answers (60 subjects). The final study sample was comprised of 2126 nurses in active employment (69.6% with a temporary contract, *n* = 1479, and 30.4% a permanent contract, *n* = 647). The mean age was 31.66 years (*SD* = 6.66), in a range of 22 to 60 years. Women with a mean age of 31.67 (*SD* = 6.65) made up 84.9% (*n* = 1805) and 15.1% (*n* = 321) were men with a mean age of 31.64 years (*SD* = 6.74).

### 2.2. Instruments

The Utrecht Work Engagement Scale (UWES; [32]) for evaluating work engagement consists of 17 items answered on a seven-point Likert-type scale. The items are distributed in three factors: Vigor (high energy and resilience levels, the desire to devote effort, not tiring easily, and persistence in the face of problems), dedication (a sense or meaning to one’s work, feeling enthusiastic and proud of one’s work, and inspired and challenged by the job), and absorption (being happily immersed in one’s work and finding it hard to stop doing it, time goes by quickly and everything around one is forgotten), offering scores on each of the scales and a total engagement scale. The reliability in this study, using the Cronbach’s Alpha for each of the dimensions was 0.84 in vigor, 0.89 in dedication, and 0.81 in absorption.

Brief Emotional Intelligence Inventory for Senior Citizens (EQ-i-20M; [33]) is an adaptation for the adult Spanish population of the Emotional Intelligence Inventory: Young Version (EQ-i-YV) by Bar-On and Parker [34]. This inventory is comprised of 20 items with four answer choices on a Likert-type scale. They are distributed over five factors: Intrapersonal, interpersonal, stress management, adaptability, and mood. In this study, the reliability indices were adequate, finding a Cronbach’s alpha of 0.90 in intrapersonal, 0.75 in interpersonal, 0.82 in stress management, 0.82 in adaptability, and 0.87 in mood.

### 2.3. Procedure

The questionnaires were implemented in 2017 on a Web platform which enabled the inclusion of control questions to detect random answers by participants, who were then eliminated from the database. Before filling in the instruments, subjects were informed of the confidentiality and anonymity of their answers. This study was approved by the Bioethics Committee of the University of Almería (Ref: UALBIO2017/011).

### 2.4. Data Analysis

First, a Pearson’s correlation coefficient analysis was performed to find a matrix of relationships between the variables in the study. Then, to know whether there were any significant differences between men and women, the Student’s *t* test for independent samples was applied for each of the dimensions of engagement. The effect size was calculated using Cohen’s *d*.

To examine how the predictor variables (emotional intelligence: Intrapersonal, interpersonal, stress management, adaptability, and mood) were related to the criterion variables (Engagement: vigor, dedication, and absorption) a stepwise multiple linear regression analysis was carried out for each of the two sex groups.

Finally, a moderation analysis was performed, using the PROCEESS macro for SPSS [35], with bootstrapping with coefficients estimated from 5000 bootstraps. Before computing the models, the moderating variable (dichotomous) was recoded as a dummy variable (men: 0 = −0.5; women: 1 = 0.5).

## 3. Results

### 3.1. Emotional Intelligence and Engagement by Sex

**Figure 1:**
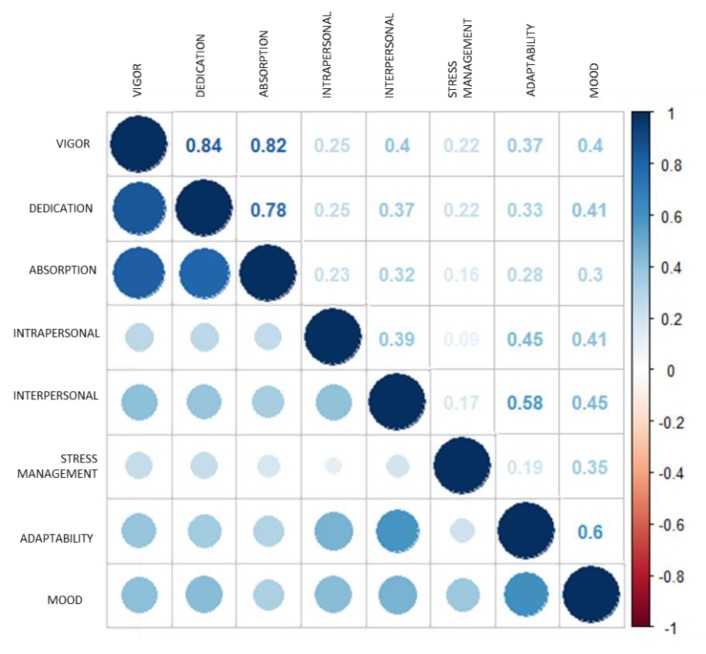
Components of emotional intelligence and engagement. Correlation matrix.

Figure 1 shows the correlation matrix of the components of emotional intelligence and engagement, for both the total sample and for men and women.

As observed, vigor correlated positively with all the components of emotional intelligence (intrapersonal: *r* = 0.25, *p* < 0.001; interpersonal: *r* = 0.40, *p* < 0.001; stress management: *r* = 0.22, *p* < 0.001; adaptability: *r* = 0.37, *p* < 0.001; and mood: *r* = 0.40, *p* < 0.001).

The dedication factor of engagement had positive correlations with intrapersonal (*r* = 0.25, *p* < 0.001), interpersonal (*r* = 0.37, *p* < 0.001), stress management (*r* = 0.22, *p* < 0.001), adaptability (*r* = 0.33, *p* < 0.001), and mood (*r* = 0.41, *p* < 0.001).

Meanwhile, the correlations established between the absorption factor and the emotional intelligence components were also positive (intrapersonal: *r* = 0.23, *p* < 0.001; interpersonal: *r* = 0.32, *p* < 0.001; stress management: *r* = 0.16, *p* < 0.001; adaptability: *r* = 0.28, *p* < 0.001; and mood: *r* = 0.30, *p* < 0.001).

Furthermore, comparing the groups based on the sex variable, statistically significant differences were observed between men and women in three factors of engagement: Vigor (*t* = −3.13, *p* < 0.01, d = 0.19), dedication (*t* = −2.84, *p* < 0.01, d = 0.17), and absorption (*t* = −3.53, *p* < 0.001, d = 0.21). In all cases, the women (vigor: *M* = 3.87, *SD* = 0.76; dedication: *M* = 4.09, *SD* = 0.77; absorption: *M* = 3.55, *SD* = 0.78) had significantly higher scores than the men (vigor: *M* = 3.72, *SD* = 0.80; dedication: *M* = 3.94, *SD* = 0.88; absorption: *M* = 3.38, *SD* = 0.84).

### 3.2. Emotional Intelligence Components as Predictors of Engagement in Nursing Professionals by Sex

Table 1 shows the models that resulted from the regression analysis for the vigor dimension, taking sex as the selection variable and the option “male”.

In the vigor dimension, the third model explained 23.7% (*R*^2^ = 0.23) of the variance. In this case, the Durbin–Watson *D* confirmed the validity of the model (*D* = 1.96). The *t* test detected an association with a probability of error below 0.5 for all the variables included in the model: Mood, interpersonal, and intrapersonal. According to the values found for the standardized coefficients, the mood variable was shown to be a stronger predictor of vigor in men. In view of the tolerance and VIF indicators in this case, collinearity of variables may be assumed to be absent.

Two models resulted from the regression analysis for dedication, the second of which explained 24.5% of the variance (*R*^2^ = 024). The Durbin–Watson *D* confirmed the model’s validity (*D* = 2.00). The association between the variables has a probability of less than 0.05, both for mood and for the interpersonal factor, which are the variables included in the model. According to the standardized coefficients, mood is the variable with the highest explanatory value. Absence of collinearity of the variables may be assumed, as the tolerance was high and VIF low.

The results found for Absorption provided three models, of which the third explained 15.3% of the variance (*R*^2^ = 0.15). To confirm model validity, independence of residuals was analyzed. The Durbin–Watson *D* showed a *D* = 1.95, which confirmed absence of positive or negative autocorrelation. The *t* was associated with a probability of error below 0.05 in the variables included in the model (interpersonal, mood, and intrapersonal). The standardized coefficients revealed that the variable with the highest explanatory value in the equation is the interpersonal factor. In this case, according to the tolerance and VIF indicators (see Table 1), absence of collinearity between the variables in the model may be assumed.

Using sex as the selection variable and “female” as the option, the regression models found for each of the dimensions of engagement were those shown in Table 2.

In the vigor dimension, there were four models, the last of which explained a variance of 23% (*R*^2^ = 0.23). Model validity was determined by the Durbin–Watson *D* of 1.96. The association between variables had a probability below 0.05 for all the variables included in the model. According to the standardized coefficients, the interpersonal factor had the most explanatory value. Absence of collinearity of variables may be assumed given the tolerance and VIF indicators.

For dedication, as shown in the table, the regression analysis suggested three models. In the third, the explained variance was 21.5% (*R*^2^ = 0.21) and the *D* = 1.92 confirmed model validity. The *t* detected an association of variables with a probability of error below 0.05 for all the variables in the model: Mood, interpersonal, and stress management. The first was the strongest predictor of dedication among women. According to the values found in the tolerance and VIF indicators (see Table 2), absence of collinearity between the variables in the model may be assumed.

Finally, for the absorption dimension of engagement, the regression analysis resulted in four models for the group of women. In the fourth model, the interpersonal, mood, stress management, and intrapersonal factors were included with 13.7% (*R*^2^ = 0.13) explained variance. The Durbin–Watson *D* = 1.95, which confirmed the absence of positive or negative autocorrelation. The *t* was associated with a probability of error below 0.05 in all the variables in the model. Furthermore, the standardized coefficients revealed that the variable with the most explanatory value was the interpersonal factor. Based on the tolerance and VIF indicators, absence of collinearity between the variables in the model may be assumed.

### 3.3. Moderation Analysis of Sex in the Relationship between Emotional Intelligence and Engagement

The conditional effect of interaction of sex as the moderating variable was tested by moderation analysis with each of the emotional intelligence factors as possible predictors of the engagement dimensions.

**Figure 2:**
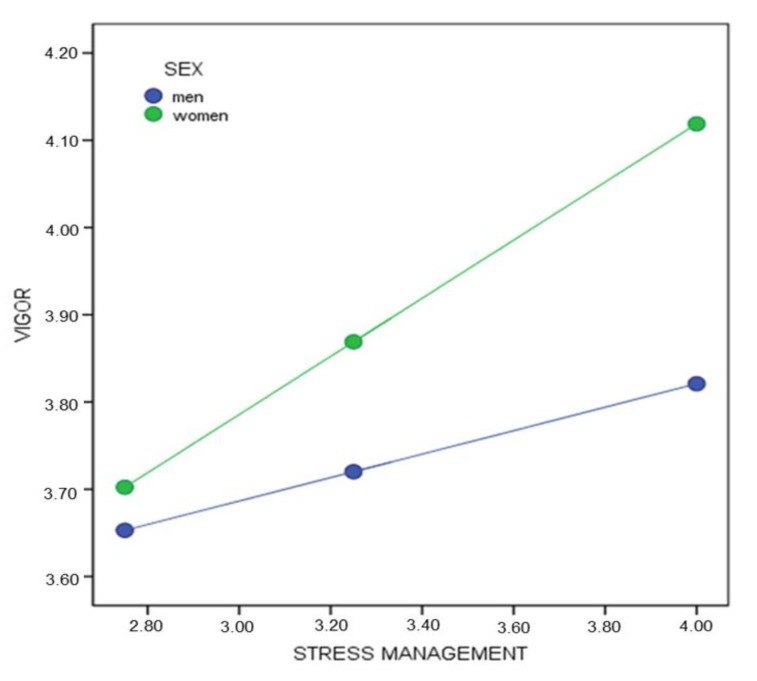
Conditional effect of stress management on vigor by sex.

First, taking vigor as the response variable and stress management as the predictor (Figure 2), a significant interaction effect was found β_(Stress management * Sex)_ = 0.19, SE = 0.08, *p* < 0.05 (95% CI = 0.042, 0.355). The conditional effect of the predictor variable on the response variable in different moderator values suggests that the simple effect of stress management is statistically significant for women β_(x→Y│M = 0.5)_ = 0.33, *p* < 0.001 (95% CI = 0.272, 0.394). However, no statistically significant differences by stress management were observed for men β_(x→Y│M = −0.5)_ = 0.13, *p* = 0.068 (95% CI = −0.010, 0.278).

**Figure 3:**
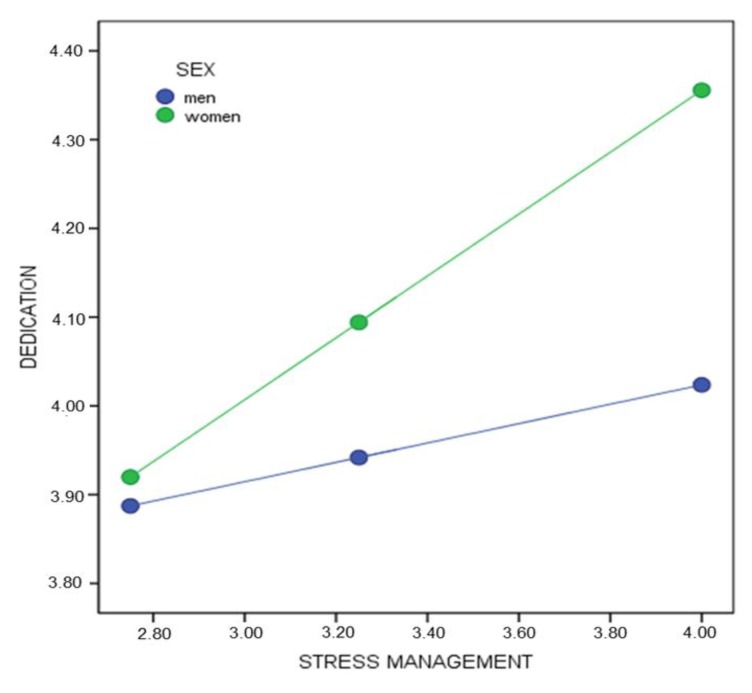
Conditional effect of stress management on dedication by sex.

Second, taking dedication as the response variable and stress management as the predictor (Figure 3), a significant interaction effect was found β_(Stress management * Sex)_ = 0.23, SE = 0.08, *p* < 0.01 (95% CI = 0.078, 0.400). The conditional effect of the predictor variable on the response variable in the different moderator values suggests that the simple effect of stress management is statistically significant for women β_(x→Y│M = 0.5)_ = 0.34, *p* < 0.001 (95% CI = 0.285, 0.411). However, no statistically significant differences by stress management were observed for men β_(x→Y│M = −0.5)_ = 0.10, *p* = 0.150 (95% CI = −0.039, 0.257).

**Figure 4:**
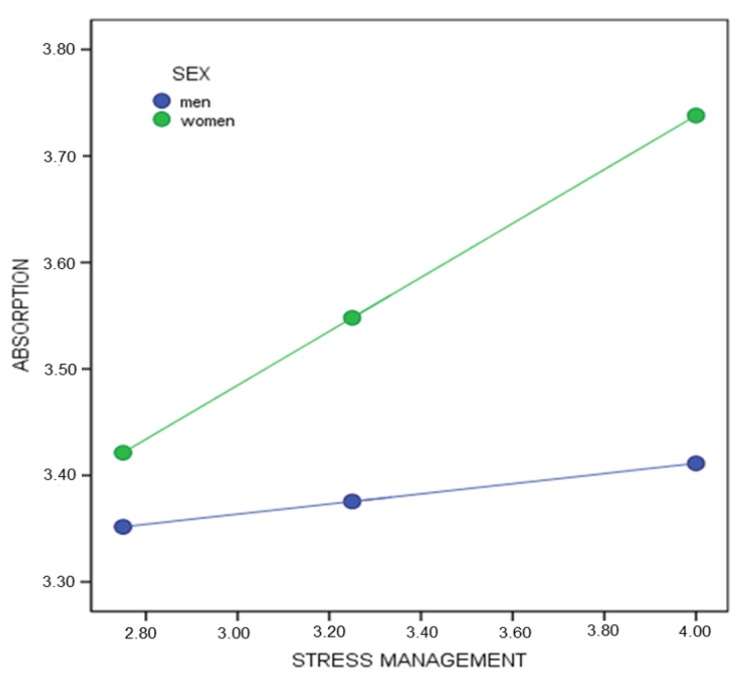
Conditional effect of stress management on absorption by sex.

Finally, with absorption as the response variable and stress management as the predictor (Figure 4), a significant interaction was found β_(Stress management * Sex)_ = 0.20, SE = 0.08, *p* < 0.05 (95% CI = 0.041, 0.370). The conditional effect of the predictor variable on the response variable on different moderator values suggests that the simple effect of stress management is statistically significant for women β_(x→Y│M = 0.5)_ = 0.25, *p* < 0.001 (95% CI = 0.189, 0.317). However, for men, no statistically significant differences were observed by stress management β_(x→Y│M = −0.5)_ = 0.04, *p* = 0.537 (95% CI = −0.104, 0.199).

## 4. Discussion

This study attempted to determine the components of emotional intelligence with the most weight in the dimensions of engagement, with sex as a potential moderator variable. In the first place, significant differences were found between sexes in engagement [25], where women had higher scores than men in all its dimensions. Thus, our first research hypothesis was confirmed, and in line with other studies, female nursing personnel scored higher on the components of engagement [26], further showing that adequate emotional management helps nurses to trust in their ability to cope with challenges and meet their goals for work successfully [28]. Based on the results in the regression models for each of the dimensions of engagement, we tested three of the hypotheses posed, finding that the emotional intelligence mood factor had the most explanatory value in the vigor and dedication components of engagement in male professionals, as it did in the study by Azimi et al. [29], who found higher scores for the mood dimension, stress management, and adaptability. Meanwhile, in women, the interpersonal factor was the strongest predictor of the vigor and absorption components. The absorption component should also be considered for possible intervention because of the characteristics of the profession [11]. Similarly, these empirical data back our hypothesis with respect to the explanatory weight of the interpersonal component of emotional intelligence in engagement in female professionals, and mood as the strongest predictor of engagement in male nurses.

Thus, based on the relationships between the two constructs [22,30,31], it becomes necessary to inquire specifically into the existence of a conditional effect of some of the components of emotional intelligence on engagement, in moderator values, in this case, the sex of nursing professionals. In this respect, we found values that support the existence of this conditional effect in stress management, which as a predictor of engagement, behaves differently in men and women. Results such as these lead us to suggest the need for a differential design in intervention for improving engagement, especially emphasizing adjustment of the treatment or program to variables which act as moderators of the effects between potential predictors and response variables.

An available repertoire of emotional resources definitely leads to acquisition of positive attitudes and skills for improving engagement, and making for better job performance [7,19] and higher job satisfaction [20,21]. The results specifically present some practical implications, such as the design and implementation of programs for preventing quitting due to demotivation and low job performance. Action focusing on worker acquisition of emotional management competencies can also be developed.

## 5. Limitations

This study had some limitations, such as the composition and distribution of the sample. In this respect, it should be pointed out that, in Spain, women are more highly represented in the healthcare profession than are men, and in nursing in particular, where over half of the professionals are women.

Secondly, data collection was based on self-report measures, so they may be considered to have the biases classically associated with this type of resource (such as the social desirability bias, which sometimes persists in spite of the guaranteed anonymity of answers). And finally, another of the limitations is that, according to the literature, a wide repertoire of variables intervening in engagement were not considered because they did not respond directly to the objectives of this study or because they were variables related to the organizational context (e.g., job climate, shifts, hospital resources, etc.). In any case, new lines of research are suggested, in which data from a diversity of sources can be included and which respond to objectives covering both individual and organizational characteristics.

## 6. Conclusions

The results derived from this study emphasize the importance of the role of the emotional intelligence dimensions in the engagement levels of nursing professionals, as well as in their health and job performance. At the same time, the differences found between men and women should be pointed out and kept in mind when designing intervention programs to improve engagement at work, job performance and benefits to the professional, patients, and the organization.

Finally, it would be advisable to increase the impact of emotional intelligence on human resource management in the healthcare environment and employ specific interventions to promote work participation.

## Figures and Tables

**Table 1:** Stepwise multiple linear regression model. Vigor, dedication, absorption (men; *n* = 321).

VIGOR	**Model**	***R***	***R*^2^**	***CorrectedR^2^***	**Change Statistics**					**Durbin–Watson**
					**Std. Error of Estimation**	**Change in *R*^2^**	**Change in *F***		**Sig. of Change in *F***	
	1	0.42	0.17	0.17	0.73	0.17	68.71		0.000	1.96
	2	0.47	0.22	0.22	0.71	0.04	20.07		0.000	
	3	0.48	0.23	0.22	0.70	0.01	4.37		0.037	
	**Model 3**			**Unstandardized Coefficients**		**Standardized Coefficients**	***t***	**Sig.**	**Collinearity**	
				***B***	**Std. Error**	**Beta**			**Tol.**	**VIF**
	(Constant)			1.26	0.25		4.98	0.000		
	Mood			0.37	0.07	0.27	4.90	0.000	0.74	1.34
	Interpersonal			0.33	0.08	0.21	3.78	0.000	0.72	1.37
	Intrapersonal			0.13	0.06	0.11	2.09	0.037	0.80	1.23
DEDICATION	**Model**	***R***	***R*^2^**	***CorrectedR^2^***	**Change Statistics**					**Durbin–Watson**
					**Std. Error of Estimation**	**Change in *R*^2^**	**Change in *F***		**Sig. of Change in *F***	
	1	0.43	0.18	0.18	0.79	0.18	72.96		0.000	2.00
	2	0.49	0.24	0.24	0.77	0.05	24.60		0.000	
	**Model 2**			**Unstandardized Coefficients**		**Standardized Coefficients**	***t***	**Sig.**	**Collinearity**	
				***B***	**Std. Error**	**Beta**			**Tol.**	**VIF**
	(Constant)			1.22	0.27		4.49	0.000		
	Mood			0.44	0.08	0.30	5.52	0.000	0.78	1.27
	Interpersonal			0.45	0.09	0.27	4.96	0.000	0.78	1.27
ABSORPTION	**Model**	***R***	***R*^2^**	**Corrected*R*^2^**	**Change Statistics**					**Durbin–Watson**
					**Std. Error of Estimation**	**Change in *R*^2^**	**Change in *F***		**Sig. of Change in *F***	
	1	0.33	0.11	0.10	0.80	0.11	39.75		0.000	1.95
	2	0.37	0.14	0.13	0.78	0.03	11.01		0.001	
	3	0.39	0.15	0.14	0.78	0.01	4.64		0.032	
	**Model 3**			**Unstandardized Coefficients**		**Standardized Coefficients**	***t***	**Sig.**	**Collinearity**	
				***B***	**Std. Error**	**Beta**			**Tol.**	**VIF**
	(Constant)			1.29	0.28		4.62	0.000		
	Interpersonal			0.33	0.09	0.20	3.41	0.001	0.72	1.37
	Mood			0.23	0.08	0.16	2.79	0.005	0.74	1.34
	Intrapersonal			0.15	0.07	0.12	2.15	0.032	0.80	1.23

**Table 2:** Stepwise multiple linear regression model. Vigor, dedication, absorption (women; *n* =1805).

VIGOR	**Model**	***R***	***R*^2^**	***CorrectedR^2^***	**Change Statistics**				**Durbin–Watson**	
					**Std. Error of Estimation**	**Change in *R*^2^**	**Change in *F***	**Sig. of Change in *F***		
	1	0.39	0.15	0.15	0.70	0.15	333.82	0.000	1.96	
	2	0.46	0.21	0.21	0.67	0.05	128.96	0.000		
	3	0.47	0.22	0.22	0.67	0.01	28.76	0.000		
	4	0.48	0.23	0.22	0.67	0.00	12.84	0.000		
	**Model 4**			**Unstandardized Coefficients**		**Standardized Coefficients**	***t***	**Sig.**	**Collinearity**	
				***B***	**Std. Error**	**Beta**			**Tol.**	**VIF**
	(Constant)			1.10	0.12		8.67	0.000		
	Mood			0.23	0.03	0.18	6.77	0.000	0.56	1.76
	Interpersonal			0.35	0.04	0.22	8.78	0.000	0.64	1.55
	Stress Management			0.16	0.03	0.12	5.42	0.000	0.86	1.15
	Adaptability			0.14	0.04	0.10	3.58	0.000	0.51	1.93
DEDICATION	**Model**	***R***	***R*^2^**	***CorrectedR^2^***	**Change Statistics**				**Durbin–Watson**	
					**Std. Error of Estimation**	**Change in *R*^2^**	**Change in *F***	**Sig. of Change in *F***		
	1	0.40	0.16	0.16	0.70	0.16	352.21	0.000	1.92	
	2	0.44	0.20	0.20	0.69	0.03	86.32	0.000		
	3	0.46	0.21	0.21	0.68	0.01	30.45	0.000		
	**Model 3**			**Unstandardized Coefficients**		**Standardized Coefficients**	***t***	**Sig.**	**Collinearity**	
				***B***	**Std. Error**	**Beta**			**Tol.**	**VIF**
	(Constant)			1.45	0.12		11.26	0.000		
	Mood			0.33	0.03	0.25	10.44	0.000	0.70	1.41
	Interpersonal			0.34	0.03	0.21	9.34	0.000	0.79	1.26
	Stress Management			0.16	0.03	0.12	5.51	0.000	0.86	1.15
ABSORPTION	**Model**	***R***	***R*^2^**	**Corrected*R*^2^**	**Change Statistics**				**Durbin–Watson**	
					**Std. Error of Estimation**	**Change in *R*^2^**	**Change in *F***	**Sig. of change in *F***		
	1	0.30	0.09	0.09	0.75	0.09	187.95	0.000	1.95	
	2	0.35	0.12	0.12	0.73	0.03	68.24	0.000		
	3	0.36	0.13	0.13	0.73	0.00	12.18	0.000		
	4	0.37	0.13	0.13	0.73	0.00	8.71	0.003		
	**Model 4**			**Unstandardized Coefficients**		**Standardized Coefficients**	***t***	**Sig.**	**Collinearity**	
				***B***	**Std. Error**	**Beta**			**Tol.**	**VIF**
	(Constant)			1.36	0.13		9.82	0.000		
	Mood			0.31	0.04	0.19	7.76	0.000	0.74	1.33
	Interpersonal			0.19	0.03	0.14	5.44	0.000	0.63	1.56
	Stress Management			0.12	0.03	0.08	3.69	0.000	0.86	1.15
	Intrapersonal			0.08	0.02	0.07	2.95	0.003	0.76	1.30
